# The mainz resilience assessment in childhood cancer (MRAcc): development of a novel age-specific patient-reported outcome measure to assess resilience in childhood cancer patients

**DOI:** 10.1186/s12885-026-15776-y

**Published:** 2026-02-26

**Authors:** Marie A. Neu, Franziska Ortmüller, Abigale L. Robinson, Elias Dreismickenbecker, Henrike Otto, Lena Wypyrsczyk, Mareike Kühn, Michèle Wessa, Oliver Tüscher, Joerg Faber

**Affiliations:** 1 Childhood Cancer Center Mainz, University Medical Center of the Johannes Gutenberg-University Mainz, Mainz, Germany; 2 Division “Cancer Survivorship and Psychological Resilience”, German Cancer Research Center (DKFZ), Heidelberg, Germany; 3 Department Cognitive and Clinical Neuroscience, Central Institute of Mental Health (CIMH), Mannheim, Germany; 4 Department of Psychiatry, Psychotherapy and Psychosomatic Medicine, University Medical Center Halle, Halle (Saale), Germany; 5 German Center for Mental Health (DZPG), Site Halle-Jena-Magdeburg, Halle (Saale), Germany; 6 Department of Psychiatry and Psychotherapy, University Medical Center of the Johannes Gutenberg-University Mainz, Mainz, Germany; 7 Leibniz Institute for Resilience Research, Mainz, Germany

**Keywords:** Childhood cancer, Pediatric Oncology, Resilience, Mental health, Stressor reactivity, Psychosocial Assessment, Patient-reported outcome measure, Supportive Care

## Abstract

**Background:**

During intensive cancer treatment, children, adolescents and young adults are exposed to numerous toxicities and psychosocial stressors that can cause psychosocial distress and impair mental health. The maintenance or rapid recovery of mental health during and after exposure to significant stressors has been defined as resilience. To date, resilience research has focused primarily on cross-sectional assessment of specific, trait-like resilience factors and concepts in long-term survivors of childhood cancer, typically omitting the influence of context-specific biopsychosocial stressors and resilience dynamics throughout treatment. Little is known about outcome-based resilience and mental health resources in childhood cancer patients undergoing cancer treatment. In addition, specific instruments for age-appropriate assessment of resilience in childhood cancer patients are lacking. To address this gap, within the EU Horizon 2020-funded FORTEe project, we developed a novel self-report instrument for longitudinal assessment of resilience in children, adolescents, and young adults with cancer, featuring age-appropriate items tailored to their specific contexts.

**Methods:**

An interdisciplinary team of psychologists, psychiatrists and pediatric oncologists developed an age-appropriate self-report instrument to assess resilience longitudinally in children, adolescents, and young adults undergoing cancer treatment. Following current resilience research frameworks, resilience is defined as the ratio of changes in mental health problems to stressor exposure. Accordingly, the measure comprises two domains: mental health problems (anxiety, depression, distress, fatigue) and stressor exposure (daily hassles, cancer-related stressors), with stressors rated for both frequency and intensity.

**Results:**

The Mainz Resilience Assessment in Childhood Cancer (MRAcc) consists of three age-specific versions (children 5–11 years, adolescents 12–17 years, young adults 18–21 years), each including the sections: 'Emotions & Distress', 'Fatigue', and 'Situations & Experienced Stress'. It is available in German and English and uses either five-point-Likert scales or visual analogue scales presented as thermometers.

**Conclusion:**

The MRAcc is a novel instrument designed to provide age-appropriate measurement of resilience in children, adolescents and young adults with cancer. It reflects the unique stressors and psychological challenges of this population and allows for longitudinal assessment throughout treatment and follow-up. Future validation studies are required to establish its psychometric properties and evaluate its utility in resilience research and clinical practice.

**Supplementary Information:**

The online version contains supplementary material available at 10.1186/s12885-026-15776-y.

## Introduction

### Stressors in pediatric oncology and the imperative to measure resilience

Children and adolescents undergoing intensive cancer therapy experience numerous disease- and treatment-related toxicities and psychosocial stressors. These include invasive procedures, high-dose chemotherapy, prolonged hospitalization and disruption of normal peer, school and family routines. These cumulative challenges predispose young patients to psychosocial distress, and putting them at increased risk for mental health impairments. Maintaining or rapidly restoring mental health during and after exposure to such stressors is defined as resilience, a dynamic process fostering treatment adherence, health-related quality of life, and long-term psychosocial outcomes [1, 2].

Although five-year survival rates now exceed 85 percent in high-income settings, the annual burden of childhood cancer remains considerable: Globally more than 380,000 individuals aged 0 to 19 receive a cancer diagnosis annually [3, 4]. Despite growing interest in psychosocial outcomes, resilience research has focused primarily on long-term survivors [5, 6], leaving a critical knowledge gap regarding resilience processes and mental health resources during active treatment. Furthermore, conceptualizing resilience as mental health related to individual stressor exposure and its respective operationalization, remains rare in cancer research in general and particularly in children and adolescents. In that regard, no age-appropriate, disease-specific instrument exists to assess resilience in children and adolescents undergoing cancer treatment.

### Understanding resilience

Resilience has long been debated as a scientific construct, with early critiques highlighting definitional ambiguities and challenges in measurement. However, more recent theoretical research frameworks proposed a new perspective, reframing resilience as a dynamic, outcome-oriented process and advocating for prospective, longitudinal designs that directly link quantified stressor exposure to changes in mental health [2, 7]. Resilience is defined here as the ability to maintain or rapidly recover mental health during and after exposure to substantial stressors, operationalized prospectively by linking quantified stressor load to changes in symptoms. [1, 2]. Similarly, Luthar and colleagues (2000) argued that resilience is not a fixed trait or mere positive adjustment, but rather the process of successfully adapting to significant adversity, necessitating clear operational definitions and direct mapping of risk exposure and adaptive outcomes [8]. A recent scoping review of resilience in children with chronic illnesses further highlights its multidimensional nature, distinguishing personal traits, psychosocial functioning, and disease-specific factors [9], and emphasizing the need for age- and disease-specific measurement tools.

In line with these conceptual and taxonomic foundation, Kalisch and colleagues introduced the Frequent Stressor and Mental Health Monitoring (FRESHMO) paradigm [1]. This operationalizes resilience by repeatedly assessing stressor load and mental health over time. In this framework, stressor exposure is sampled at regular intervals and mental health problems are measured concurrently. Based on van Harmelen and colleagues’ residualization idea, a stressor-reactivity (SR) score is then calculated as the residual deviation from the (in-group) normative regression of mental health on stressor load [1, 10]. Repeated assessment of SR scores allows resilience to be examined as a dynamic process over time.

When applied to pediatric oncology, this paradigm involves frequently measuring cancer treatment-specific stressors, such as pain severity, health-related anxiety, and barriers to discussing the illness, alongside everyday challenges, such as peer isolation or missed schooling. Concurrently, standardized assessments would need to capture mental health issues (e.g. depressive symptoms and anxiety) in order to compute individual resilience indices. This approach enables identification of patients who maintain or rapidly regain psychological well-being despite the considerable burdens of cancer treatment as well as those at risk of persistent distress who may benefit from targeted psychosocial interventions. Furthermore, such longitudinal assessment may allow to identify resilience mechanisms relevant and potentially specific to this target population.

### Current state of resilience research in childhood cancer

Over the past decade, considerable effort has been devoted to characterizing resilience and understanding and supporting the psychosocial adaptation of children with cancer and their families. However, significant gaps remain in our ability to assess these processes in real time and across the dynamic phases of treatment. To date, most studies have focused on long-term survivors [5, 6], family members, parents or other caregivers [11, 12], and siblings [13, 14], employing retrospective or end-of-treatment assessments that do not allow for the prospective assessment of the potential fluctuations in resilience-related outcomes during active cancer treatment. Moreover, the conceptual and methodological approach to assessing resilience as mental health related to individual stressor exposure, as outlined above, is not yet common in cancer research and represents an important research gap.

Although resilience in children with cancer has been increasingly studied, most existing research relies on retrospective or post-treatment assessments, limiting insight into dynamic developments during intensive treatment [15].

A recent systematic review highlighted that existing self-report coping measures for children and adolescents with cancer vary considerably in psychometric quality, with many instruments failing to support reliable measurement of resilience processes [16].

Psychometric evaluations have similarly emphasized the lack of validated, cancer-specific self-report tools for pediatric patients, noting frequent reliance on proxy reporting and limited sensitivity to intra-treatment fluctuations [16].

To date, only a limited number of systematic reviews have examined psychosocial and family-level factors associated with adaptation in childhood cancer [17, 18]. While these reviews provide valuable insights into coping and adjustment mechanisms, they also reveal that most existing studies relied on discrete outcome assessments conducted post-treatment or during aftercare, which limits our understanding of how resilience and coping evolve dynamically throughout cancer treatment [17, 18].

Complementing these quantitative findings, qualitative studies of everyday stressors [19], ranging from treatment side effects and routine disruption to social isolation, highlight that children employ a mix of problem-focused, emotion-focused, and avoidance coping strategies. However, no existing measure captures how these strategies fluctuate in response to specific treatment milestones.

Taken together, these findings highlight the lack of suitable measurement tools, especially those that are child-friendly, cancer-specific and incorporate the patient’s perspective and stressor-load, while being capable of dynamically capturing changes throughout and between treatment phases.

### Need for a new instrument

As highlighted above a self-report tool simultaneously assessing resilience prospectively during active cancer treatment, integrating both quantifiable stressor exposure and concurrent mental health outcomes and covering the developmental range from early childhood through adolescence to young adulthood is highly needed.

A self‐report approach is particularly vital in pediatric oncology, as children and adolescents offer unique insights into their own emotions, coping strategies, and perceived stressors, which cannot be fully captured by parent or clinician reports. Enabling patients to articulate their experiences promotes engagement, boosts the instrument’s real-life relevance and reduces the risk of adult‐centric bias in measurement [20, 21]. Moreover, age‐appropriate self‐report items can be tailored to different cognitive and linguistic stages, thereby ensuring that the instrument remains accessible and meaningful across a broad developmental spectrum [21].

To address these challenges, a novel patient-reported outcome measure (PROM) must be capable of being applied across diverse cancer types and treatment regimens; designed for longitudinal use, allowing for repeated administration at clinically relevant intervals; and flexible in format, permitting both paper‐and‐pencil and electronic delivery to accommodate varying clinical settings and patients’ preference. Such an instrument would not only fill a methodological gap but also enable clinicians and researchers to track resilience trajectories, identify early signs of psychological vulnerability, and tailor interventions dynamically throughout treatment.

We developed the Mainz Resilience Assessment in Childhood Cancer (MRAcc), a brief age-specific self-report instrument that quantifies stressor exposure and concurrent mental health problems to enable longitudinal estimation of stressor reactivity during active cancer treatment. The aim of this study is to present the rationale, development process and final structure of the MRAcc.

## Materials and methods

### Conceptual framework and operationalization of stressor reactivity

The H2020 EU-funded FORTEe research project “Get strong to fight childhood cancer – An exercise intervention for children and adolescents undergoing anti-cancer treatment” (NCT05289739) provided the overarching context and infrastructure for PROM administration [22], including participant recruitment and longitudinal data collection, but this manuscript focuses exclusively on the instrument’s rationale and design and does not report intervention outcomes or trial results.

The MRAcc was developed to operationalize resilience as the dynamic interplay between stressor exposure and psychological health, drawing on the Frequent Stressor and Mental Health Monitoring (FRESHMO)-paradigm that employs frequent monitoring of stressor exposure and psychological health to derive stressor-reactivity [1]. Stressor-reactivity is a metric that contextualizes mental health problems by taking the individual level of stressor exposure into account, conceptually indicating whether mental health problems are higher or lower than would be expected given the experienced adversity [1]. Building on Harmelen et al.’s (2017) and Kalisch et al.’s (2021) framework, resilience is quantified by a stressor-reactivity metric that expresses the ratio of changes in mental health problems from before to after stressor exposure [1, 10]. Thus, the instrument is structured into two key domains—mental health problems (anxiety, depression, distress, fatigue) and stressor exposure (daily hassles and cancer-specific challenges)—with each stressor evaluated according to both frequency and intensity.

The four domains included in the Mental Health Problems sub-score were selected as they represent the most common and burdensome subjectively reported psychological and cognitive symptoms in pediatric oncology, which are developmentally prevalent, clinically relevant during treatment, and suitable for repeated self-report across childhood, adolescence, and young adulthood. Fatigue, in particular, is a highly prevalent and clinically significant symptom in pediatric oncology, with well-documented fluctuations across treatment phases [23, 24]. Fatigue was included to capture the patient-reported experience of tiredness, weakness, increased sleep, and concentration difficulties, reflecting the subjective symptom burden of fatigue and its relevance for psychological functioning, without aiming to assess underlying biological mechanisms. Importantly, fatigue is conceptualized here as a biopsychosocial symptom and as one component of the overall mental health burden contributing to resilience outcomes, rather than as an external determinant of resilience or a driver of potential resilience fluctuations. Other psychiatric domains (such as psychotic, substance-related, or externalizing disorders) are rare in this age group/population or not appropriate for brief, age-appropriate self-report assessment during intensive cancer treatment, and were therefor not included as core indicators [25–27]. For the stressor exposure sub-score, three daily hassles and three cancer-specific items were chosen to reflect both developmental and illness-related challenges, providing broad coverage while keeping the number of items feasible and age-appropriate.

### PROM development process

#### Item generation, expert review, translation, cognitive pretesting

An initial pool of items was compiled from validated measures addressing anxiety, depression, distress, fatigue, and was supplemented by novel items addressing daily hassles and cancer-specific stressors. A multidisciplinary panel comprising seven experts, including one pediatric oncologist and two physicians with more than ten years of experience in pediatric oncology care, one psychiatrist who is a neurologist and psychotherapist, one psychologist who is a psychotherapist, and two exercise scientists assessed each item for conceptual relevance, clarity and age-appropriateness. This led to the iterative revision of wording and response formats.

The PROM development included age-specific versions of the PROM (for children aged 5–11, adolescents aged 12–17, and young adults aged 18–21). Throughout the development process, each item was drafted in both German and English from the outset and then underwent multiple rounds of back-translation checks by bilingual team members to avoid subtle semantic discrepancies. Native-speakers were then convened in iterative review workshops to decide on the phrasing and conceptual alignment. Once consensus had been reached on the German-English source versions, the final instrument was professionally translated (into French, Italian, Slovenian, Danish and Spanish) in accordance with the ISO 17100 standard for translation services, which includes qualified translation, independent revision, and quality assurance procedures. This was followed by a review of the translated versions by native speaking regional clinical collaborators to ensure fidelity of meaning and appropriateness for use in the respective clinical contexts. Where necessary, feedback from this review was discussed in reconciliation meetings involving the regional collaborators and members of the expert panel, supporting readiness for multinational implementation. No major cultural adaptations were identified during this process.

A cognitive pretesting was conducted with eight childhood cancer patients, covering the full age range to capture developmental diversity, with the aim of ensuring that the PROM design was grounded in the perspectives and language of the intended end users. At the time of pretesting, participants were undergoing active cancer treatment, including intensive treatment or maintenance therapy, or were in early survivorship. Participants completed the instrument and subsequently provided structured feedback, including open-ended debriefing questions and written comments addressing overall comprehensibility, clarity of wording, and acceptability of the PROM. The feedback indicated that the items were generally well understood and acceptable across age groups, and no substantial modifications to the instrument were required based on the pretesting.

## Results

### Final instrument structure

The MRAcc consists of three age-specific versions for children (5–11 years), adolescents (12–17 years), and young adults (18–21 years). Beyond visual response formats, age-specific adaptations also include developmentally tailored item wording, contextualized stressor content, and age-appropriate instructional framing. Specifically, affect-related items use simplified wording in the children’s version (e.g., “How sad have you been?”) and more differentiated formulations in adolescents and young adults (e.g., inclusion of “depressed”). Stressor items are adapted to age-typical life contexts, such as school or kindergarten in children, education in adolescents, and professional training or work in young adults. In addition, response anchors and instructions are simplified and more supportive in the children’s version, while adolescents and young adults receive more standardized formulations.

**Fig. 1:**
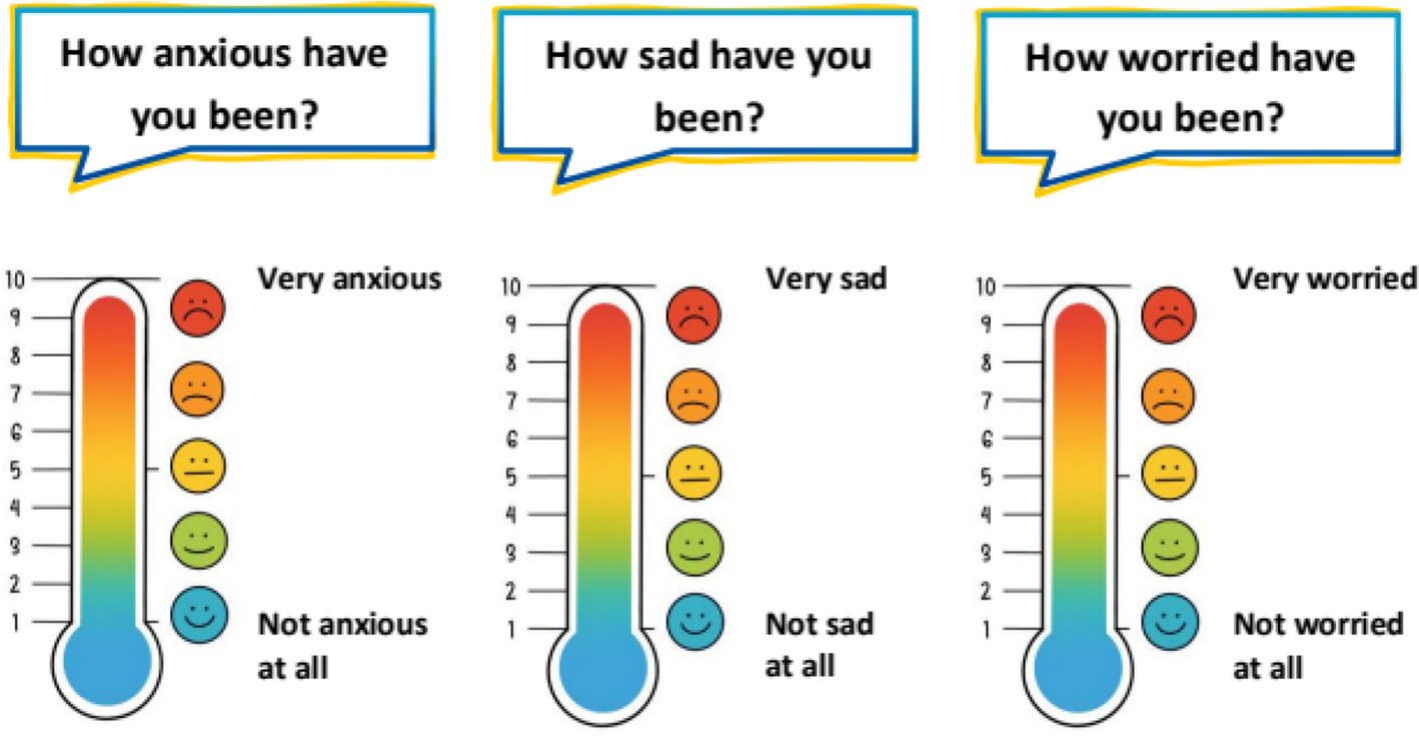
“Emotion and Stress” thermometers—Single‐item visual analogue “thermometers” (with colored smiley options for younger children)

**Fig. 2:**
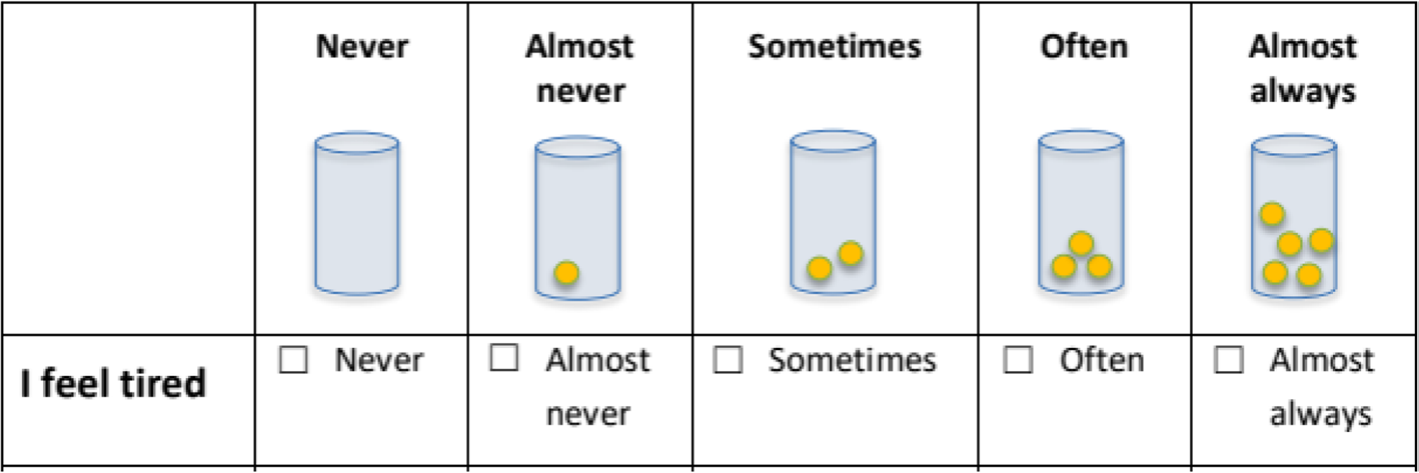
Visual five-point Likert scale for younger children (5 to 11 years), using containers filled with increasing numbers of yellow balls to indicate frequency

**Fig. 3:**
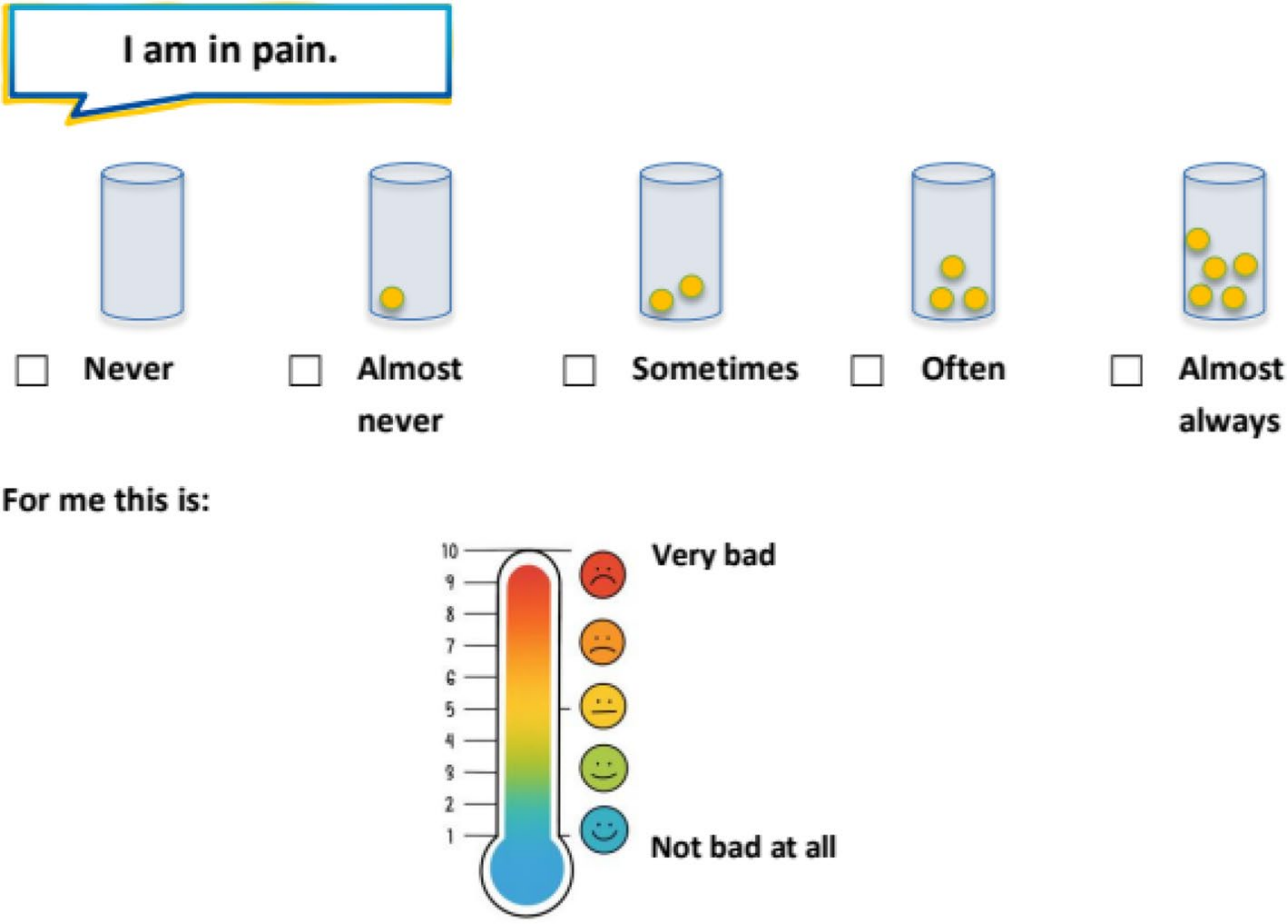
Dual-format response scale for younger children: frequency indicated by containers with yellow balls, and intensity rated via a 10-point thermometer with a color-smiley gradient

The MRAcc is available in German, English, French, Italian, Slovenian, Danish, and Spanish. The instrument yields two sub-scores:

1. Mental Health Problems sub-score (7 items total), encompassing four domains (anxiety, depression, distress, fatigue).
  “Emotion and Stress” thermometers (3 items): Single‐item visual analogue “thermometers” (with colored smiley options for younger children) are used to rate anxiety, depression and distress. Respondents indicate their current level on a vertical color gradient (blue = lowest, red = highest) marked 1–10. Example prompts include “How anxious have you been?”, “How sad or depressed have you been?”, and “How worried have you been?”. This approach is based on the Emotion Thermometers, a validated set of visual analogue scales designed to assess emotional burden in clinical and general populations [28]. For younger children, a version with colored smiley faces (Fig. 1) is used to enhance comprehensibility.
  Fatigue items (4 items): These statements are rated on a five-point Likert scale (never, almost never, sometimes, often, and almost always): “I feel tired,” “I feel weak,” “I sleep a lot,” and “It’s hard for me to concentrate”. Fatigue was assessed using multiple items to capture its heterogeneous and multidimensional subjective manifestations. In order to ensure equal weighting of fatigue relative to the single-item domains (anxiety, depression, and distress), the four fatigue items are aggregated into a mean score, such that the fatigue domain contributes one composite value to the mental health problems sub score. This approach prevents disproportionate weighting of fatigue while improving content coverage within the domain. For younger children (ages 5–11), the Likert scale is presented using a visual format with containers filled with different numbers of yellow balls (Fig. 2), allowing for age-appropriate and intuitive rating of frequency.
2. Stressor Exposure (“Situations & Experienced Stress” Thermometer) sub-score (6 items total), covering six stressors.
  Item content: Three daily-hassle items (“There are arguments in my family.”, “I am worried about school or my education.”, “I have trouble with others.”) and three cancer-specific stressors (“I am in pain.”, “I am worried about my illness.”, “I find it difficult to talk about my illness.”).
  Response format: Each item is presented twice, first as a five-point frequency question (never, almost never, sometimes, often, almost always), and immediately next to it as an intensity thermometer ranging from 1 to 10, using the same colored (smiley) gradient. For younger children, the frequency scale is visualized using containers filled with different numbers of yellow balls, while the intensity scale is represented by a vertical thermometer with a color gradient from blue to red and corresponding smiley faces. This dual-format approach supports both cognitive accessibility and emotional expressiveness (Fig. 3). Frequency (0–4) and intensity (1–10) ratings of each stressor were multiplied and the average stressor exposure of all six stressors was calculated. The multiplication of frequency and intensity scores represents a novel operationalization within the MRAcc. This approach was chosen for conceptual and practical reasons to capture overall stressor load as a function of both stressor frequency and intensity and will be evaluated in future psychometric validation analyses.

To ensure unity across the different scales, each item’s score is transformed to a 1–100 scale.

By combining visual analogue (smiley) thermometers with familiar Likert scales, the MRAcc aims to balance sensitivity to momentary affective states with reliable measurement of fluctuating symptom burden and stressor exposure.

Sample item layouts are illustrated in Figs. 1, 2 and 3. The full age-specific PROM can be found in Additional file 1.

### Scoring interpretation and visualization

The MRAcc generates two separate sub-scores that can optionally be combined into a ratio for interpretive guidance, though primary emphasis is placed on joint plotting as per the FRESHMO paradigm [1]. Rather than collapsing sub-scores into a single ratio, resilience is then visualized by plotting Mental Health Problems against Stressor Exposure in a two-dimensional space, consistent with the FRESHMO framework [1].

The transformation and aggregation procedures applied in the MRAcc were chosen based on conceptual and pragmatic considerations. Item scores were transformed to a common 1–100 scale to harmonize different response formats and support intuitive interpretation.

Numeric thermometer scales are clinically well established in pediatric, adolescent, and adult populations and were therefore used in the adolescent (12–17 years) and adult (≥ 18 years) versions of the instrument. For children aged 5–11 years, an age-adapted thermometer format was used to support comprehension and reliable self-report.

### Detailed transformation and aggregation

- **Mental Health Problems sub-score:**
  - Three thermometer items: Anxiety, depression, distress are each rated via a 1–10 colored (smiley) thermometer and were transformed to 1–100 scale.
  - Four fatigue items: Each statement (“I feel tired,” “I feel weak,” “I sleep a lot,” “It is hard for me to concentrate”) is rated on a five-point Likert scale (never to almost always) and was then transformed to a 1–100 scale. In order to achieve equal weighting across the mental health domains of anxiety, depression, distress, and fatigue, the four items assessing fatigue were averaged into a single composite score.
  - Aggregation: To calculate the mental health problems score, the four transformed scores of anxiety, depression, distress and fatigue were averaged, yielding a 1–100 mental health problems sub-score.
- **Stressor Exposure sub-score:**
  - Each of the six assessed stressors was measured based on both its frequency (using a five-point Likert scale) and its perceived intensity (rated on a 1–10 scale). To ensure comparability across items, both scales were transformed to a 1–100 scale.
  - Frequency and intensity scores for each stressor were multiplied and normalized by dividing the product by 100. To calculate the overall stressor score, the normalized values for all six stressors were summed and averaged, yielding a 0.01–100 stressor exposure sub-score.

### (Smiley) Thermometer coding

In the child version (5–11 years), thermometer items are presented with both numeric anchors (1–10) and five color-coded facial expressions (smileys). If a numeric value is selected, this value directly enters the score. If a smiley is selected, responses are coded to preserve the ordinal progression of perceived intensity conveyed by the visual anchors (red = 10, orange = 7, yellow = 5, green = 3, blue = 1). Selection of the bulb below the lowest smiley is coded as zero.

This coding scheme builds on established visual analogue and emotion thermometer approaches and was selected to preserve ordinal intensity information without implying interval-level measurement, while enabling harmonized scoring across age groups.

### Ratio-based and regression-based perspectives on stressor reactivity

The following section introduces two complementary but distinct ways of interpreting stressor reactivity. The first, a simplified ratio-based approach, is suitable for individual-level or clinical applications. The second, a residual-based approach, reflects the regression principle underlying the FRESHMO framework (1) and serves as a conceptual reference for model-based visualization.

A preliminary interpretive ratio may be computed as

$$MRAcc Ratio=\frac{Mental Health Problems Subscore}{Stressor Exposure Subscore}$$

This simplified ratio is intended to provide a pragmatic, individual-level indicator of relative stressor reactivity that can be applied without the need for regression analysis. While conceptually inspired by the regression-based stressor reactivity score described above, which reflects a group-level statistical association, the ratio does not rely on population parameters or residual estimation and therefore allows interpretation at the single-case level, for example in clinical settings.

Values below 1 suggest a relatively **low symptom burden despite higher exposure** (indicating adaptive or resilient functioning), while values above 1 suggest a **greater symptom burden at lower exposure** (indicating increased stressor reactivity or potential non-resilient functioning).

However, in practice, interpreting a single ratio can be misleading, as it may mask whether low stressor reactivity results from a high stressor load, a low symptom burden, or both. For this reason, ratio-based scores should not replace a visual inspection of stressor and symptom levels plotted together.

Thus, the recommended primary approach remains the two-dimensional plot, which preserves these distinct contributions of mental health and stressor exposure and allows for a visual assessment of stressor reactivity patterns. In line with the regression-based rationale of the FRESHMO framework [1], this visualization offers an intuitive representation of stressor reactivity, highlighting individual deviations from the normative stressor–symptom association.

**Fig. 4:**
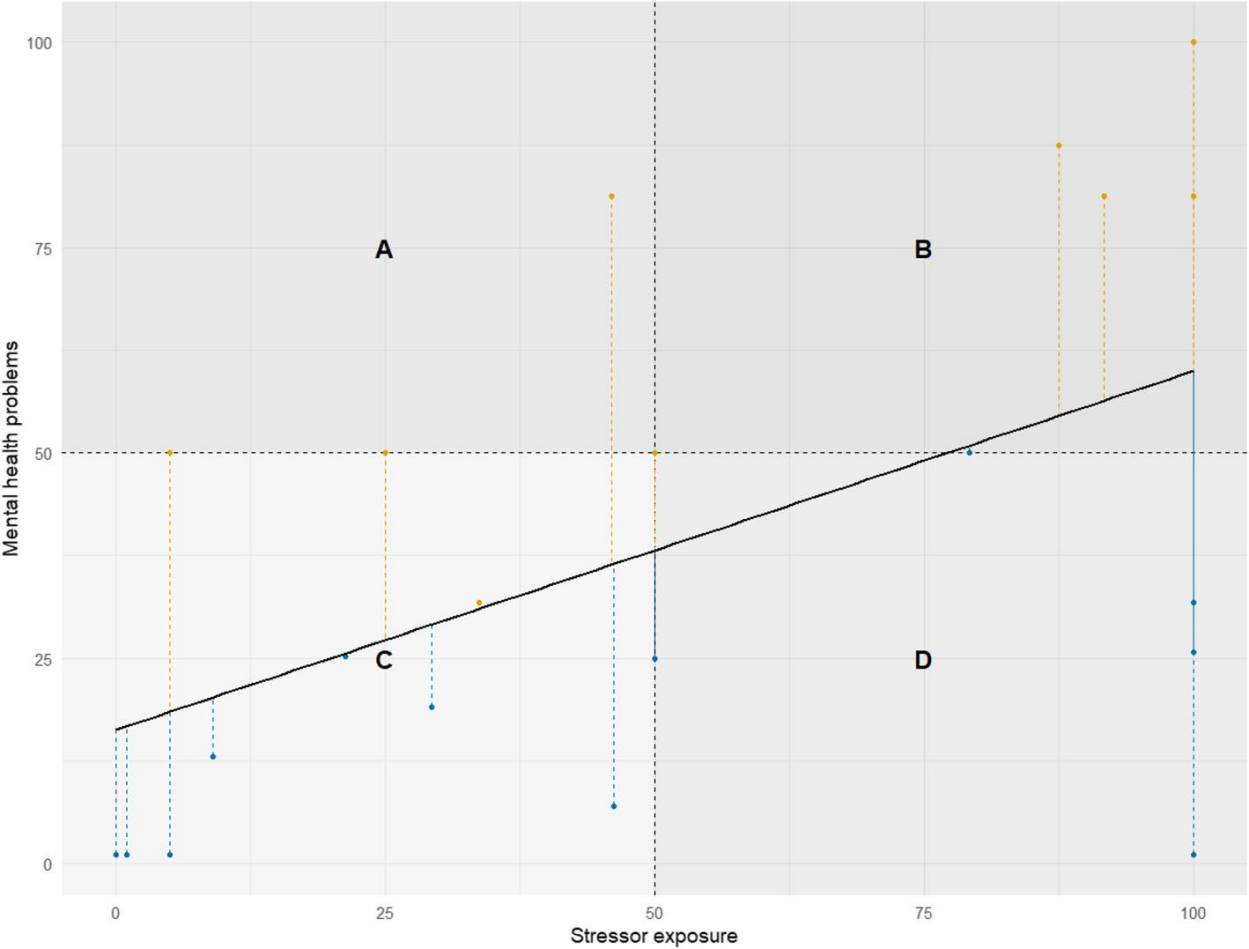
Conceptual illustration of stressor-symptom profiles based on simulated data, demonstrating the regression-based rationale of the FRESHMO framework [1]. The x-axis represents overall stressor exposure, and the y-axis represents the level of mental health problems. The black line depicts the hypothesized association between stressor exposure and mental health problems. Colored vertical dashed lines indicate deviations from this model (residuals), with downward deviations (blue) indicating lower-than-expected symptom levels given the level of stressor exposure (adaptive or reduced stressor reactivity), and upward deviations (orange) indicating higher-than-expected symptom levels (non-adaptive or increased reactivity). The four labeled regions illustrate prototypical patterns of functioning: **A** low stressor exposure with high symptoms (vulnerable pattern), low stressor exposure with high symptoms (vulnerable pattern), **B** high stressor exposure with high symptoms, **C** low stressor exposure with low symptoms, and **D** high stressor exposure with low symptoms (resilient pattern)

Figure 4 provides an exemplary conceptual illustration, based on simulated data, that highlights regions of functioning based on the interaction between stressor exposure and symptom severity, supporting both cross-sectional interpretation and the analysis of longitudinal stressor reactivity trajectories [29].

### Initial interpretive guidelines for model-based visualization

The two-dimensional plot supports interpretation of stressor–symptom interactions by illustrating prototypical patterns of stressor reactivity. Four conceptual regions can be described within the plot space:

A. Low stressor exposure/high mental health problems: May reflect increased symptom vulnerability, possibly due to other unmeasured factors rather than stressor exposure.
B. High stressor exposure/high mental health problems: May indicate proportional stressor reactivity, or limited resilience under high load.
C. Low stressor exposure/low mental health problems: Typically represents a non-challenged baseline state, not informative for resilience analysis.
D. High stressor exposure/low mental health problems: May indicate adaptive or resilient functioning, reflecting low stressor reactivity.

Considering all four regions jointly is therefore crucial, as cases with proportionally high or proportionally low stressor and symptom scores may result in similar ratio values, even though only conditions involving substantial stressor exposure are informative for resilience assessment.

Interpretation focuses on deviation from the normative stressor-symptom association, rather than strict categorization into predefined zones. Profiles near the modeled regression line may indicate proportional reactivity, while marked deviations, especially those showing lower-than-expected symptoms, may suggest resilient outcomes. Visual inspection is particularly useful for longitudinal or repeated assessments, especially in clinical practice, where tracking individual stressor reactivity trajectories over time might support adaptive intervention planning.

The interpretive approaches described here are intended to provide conceptual and practical guidance for using the MRAcc in both research and clinical contexts. Formal validation of the ratio-based and regression-based interpretations, including their sensitivity to change, stability over time, and clinical utility, will be addressed in future empirical studies. In particular, planned longitudinal analyses within the FORTEe study and subsequent validation work will examine how these interpretive methods perform in relation to external clinical outcomes and established measures of mental health and stressor exposure.

### Feasibility from cognitive pretesting

In cognitive pretesting with eight participants (ages 5, 7, 10, 11, 13, 16, 18 and 21) undergoing active cancer treatment or early survivorship, all respondents reported that the items were clear, age-appropriate and acceptable in length, supporting the feasibility and content clarity of the PROM for use in the target population. Minor wording refinements were implemented for younger children to improve comprehension. These formative findings supported the usability of the age adapted layouts and informed the final instrument presented here.

### PROM administration & missing data

The MRAcc is intended for use with pediatric and young adult cancer patients via printed paper forms or electronic PDF files. PROMs should refer to a pre-defined recall period (ideally no longer than two weeks), with clear instructions that respondents report only events and experiences within this pre-defined recall period. Young children may receive reading assistance from a trained adult, provided that the assistant reads questions verbatim without interpretation.

To ensure consistent scoring, predefined rules were established to handle missing responses, specifying minimum completion requirements for calculating the mental health problems score, the stressor exposure score, and the derived stressor reactivity indicators.

Detailed instructions for handling incomplete responses can be found in Additional file 2.

## Discussion

### Relevance and innovation

Resilience in pediatric oncology remains under-assessed because most existing studies focus on outcomes such as physiological recovery or generic psychosocial well-being [30], rather than on children’s dynamic capacity to maintain or regain adaptive functioning when confronted with cancer treatment-related and general everyday stressors. Existing resilience scales omit cancer-specific challenges such as coping with cancer-related fatigue, ongoing pain or anxiety about disease progression, thereby limiting their relevance for this population. To address this unmet need, we therefore developed the MRAcc, a PROM designed to capture the unique stressors and mental health problems in young cancer patients.

Building on its strictly patient-reported perspective, the MRAcc incorporates several novel features, including cancer‐specific domains targeting disease- and treatment‐related stressors such as fatigue and pain, as well as age‐adapted response formats that combine pictorial elements and cues with traditional Likert scales.

In contrast to existing, resource-oriented resilience measures, such as the Child and Youth Resilience Measure (CYRM) [31], the Resiliency Scales for Children and Adolescents (RSCA) [32], the Adolescent Resilience Questionnaire (ARQ) [33], and the Resilience Scale for Adolescents (READ) [34], which primarily assess protective factors and individual or social resources, the MRAcc conceptualizes resilience as an outcome of adaptation to quantified stressor exposure. None of the mentioned existing instruments include direct assessments of stressor exposure, all lack disease- or cancer-specific content, and often have limited applicability to younger age groups. Further, the Brief Resilience Scale [35] assesses a general self-perceived ability to recover from stressful or adverse events (hence out-come-based resilience) but does not quantify stressor exposure either. It was originally validated in German-speaking adults aged 18 years and older and not in children and adolescents, nor are there any adaptations to age-specific or disease-related contexts available to date. The Connor-Davidson Resilience Scale (CD-RISC) [36] was similarly developed for adult populations and lacks validation in pediatric oncology or younger age groups. It measures general personal attributes related to resilience but does not include stressor assessments or cancer-relevant content, further limiting its suitability for use in children and adolescents undergoing cancer treatment. By integrating age-adapted response formats with cancer-relevant stressor and symptom assessments, the MRAcc complements existing resilience scales and provides a framework suited for longitudinal monitoring and clinically meaningful assessment in clinical practice, as well as for research applications examining resilience trajectories during active cancer treatment.

### Strengths and limitations

#### Strengths

As mentioned above, the MRAcc’s patient-centered and age-appropriate design is one of this novel PROM's key strengths, ensuring that its items are developmentally appropriate and accurately reflect the child’s perspective. This approach was reinforced through stakeholder engagement, drawing on qualitative insights from a multi-professional expert team. Integrating these two perspectives ensures that the final items more accurately reflect resilience as experienced in the pediatric oncology setting. Rigorous translation procedures were employed to facilitate broader adoption and data pooling across sites, securing conceptual equivalence across languages and enabling international, multi-center studies while mitigating the small sample sizes typical of single-site research. Finally, the MRAcc is explicitly designed for longitudinal use, allowing repeated administrations to monitor intra-individual resilience trajectories throughout treatment and to compare these patterns inter-individually across cohorts, thereby enhancing analyses of both within- and between-group change.

#### Limitations

There are several limitations to the current development phase that warrant consideration. Firstly, pre-testing of the novel PROM was conducted in a small sample of eight participants from a single center, which do not allow generalizability with respect to the relevance of the items and therefore the MRAcc must be validated in a larger cohort in future research. Formal psychometric validation, including structural validity, reliability and responsiveness analyses, is currently ongoing and will be reported in subsequent validation studies. Secondly, as the MRAcc is a self-report instrument, it is subject to recall bias and social desirability, particularly among younger children, who may be inclined to provide socially acceptable responses. However, this limitation is inherent to all self-report measures and have to be pondered against the advantages of gathering individual information from the perspective of the investigated individuals. In future applications, recall bias and social desirability may be mitigated by combining MRAcc self-reports with complementary caregiver or clinician-reported assessments, shorter recall periods, or repeated measurements over time.

#### Clinical and research implications

Integrating the MRAcc into longitudinal observational or interventional studies will allow researchers to observe resilience trajectories over time, informing the timing and nature of supportive interventions. Clinicians may use trends in individual scores or patterns of scores to identify patients who might benefit from additional psychosocial resources. Meanwhile, children and their families may receive feedback on mental health symptoms and stressor reactivity, which might promote engagement and self-management throughout the treatment journey. Furthermore, longitudinal assessment of resilience with the MRAcc will allow the identification of resilience factors and mechanisms specific to child and adolescent cancer patients, which in turn will enable and leverage the development of appropriate prevention measures targeting mental health in this population.

In clinical practice, MRAcc data may support structured decision-making by helping clinicians identify periods of increased vulnerability or reduced resilience and to tailor the timing, intensity, and focus of psychosocial support accordingly. Moreover, repeated MRAcc assessments may facilitate personalized psychosocial care by informing targeted resilience-building interventions adapted to individual stressor profiles and symptom trajectories.

## Conclusions

In summary, the MRAcc is a novel instrument designed to assess resilience in children, adolescents and young adults undergoing cancer treatment. It captures the specific stressors and psychological challenges experienced by this population and supports longitudinal assessment throughout cancer treatment. Its patient-centered design, age-appropriate response formats and multilingual readiness position it to provide valuable insights into resilience of childhood cancer patients. By enabling the joint assessment of stressor exposure and mental health outcomes, the MRAcc offers clinically and scientifically relevant information that may support longitudinal monitoring, hypothesis-driven resilience research, and informed psychosocial care during active treatment. Further validation work will, of course, be necessary to confirm the robustness of its psychometric properties and its practical usefulness in resilience research and clinical settings. Planned validation efforts include formal psychometric evaluation of reliability and construct validity, assessment of longitudinal responsiveness and sensitivity to change, and examination of clinical utility in relation to external outcomes.

## Supplementary Information

Additional file 1: Full MRAcc, including three age-specific versions (children 5–11 years, adolescents 12–17 years, young adults 18–21 years)

Additional file 2: Missing data rules

## Data Availability

The MRAcc versions and specifications for handling missing data will be available as Additional Files and in a public repository. The qualitative data from the cognitive pretesting presented in this article are not readily available because the qualitative research data contain personal information of participants and cannot be fully anonymized. In order to comply with the European and national data protection regulations applicable to this project, data access is therefore restricted.

## Abbreviations

ARQ: Adolescent Resilience Questionnaire
BRS: Brief Resilience Scale
CD-RISC: Connor-Davidson Resilience Scale
CYRM: Child and Youth Resilience Measure
FRESHMO: Frequent Stressor and Mental Health Monitoring
MRAcc: Mainz Resilience Assessment in childhood cancer
PROM: Patient-reported outcome measure
READ: Resilience Scale for Adolescents
RSCA: Resiliency Scales for Children and Adolescents
SR: Stressor-reactivity
