# Supplementary material for: The mainz resilience assessment in childhood cancer (MRAcc): development of a novel age-specific patient-reported outcome measure to assess resilience in childhood cancer patients
Source: BMC Cancer. 2026 Feb 26;26:312. doi: 10.1186/s12885-026-15776-y (PMC12952046; doi:10.1186/s12885-026-15776-y)
Supplement: Supplementary file 1 — Additional file 1: Full MRAcc, including three age-specific versions (children 5–11 years, adolescents 12–17 years, young adults 18–21 years) [file 12885_2026_15776_MOESM1_ESM.pdf]

Name:	Date:

Zentrum für Kinder- und Jugendmedizin

Mainz Resilience Assessment in Childhood Cancer

MRAcc

For children aged 5 – 11 years

Version 1.0

© 2025, University Medical Center Mainz of the Johannes Gutenberg-University Mainz, Neu, MA., Ortmüller, F., Robinson, AL., Dreismickenbecker, E., Otto, H., Wypyrsczyk, L., Kühn M., Wessa, M., Tüscher, O., Faber, J. All rights are reserved.

Funded by
the European Union

Name:	Date:

Hello! **I would love to know how you've felt in the last 2 weeks.**

That's why I've brought my thermometers with me to help you.

A 10 means that you have felt like this a lot. A 1 means that you have not felt like this at all. You can mark your answer on the thermometer underneath each question. There is no right or wrong answer.

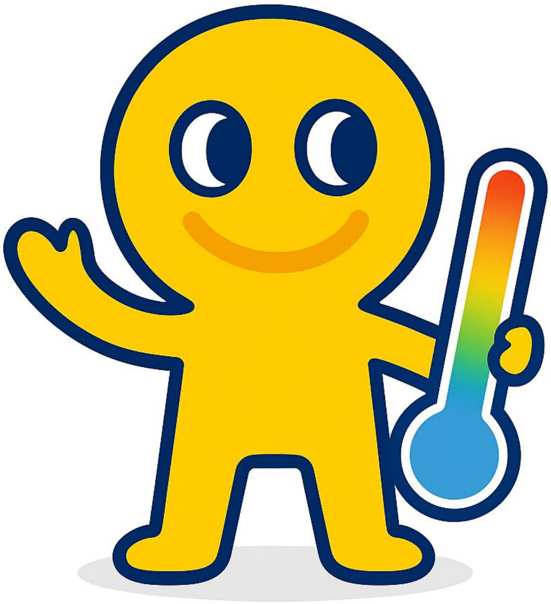

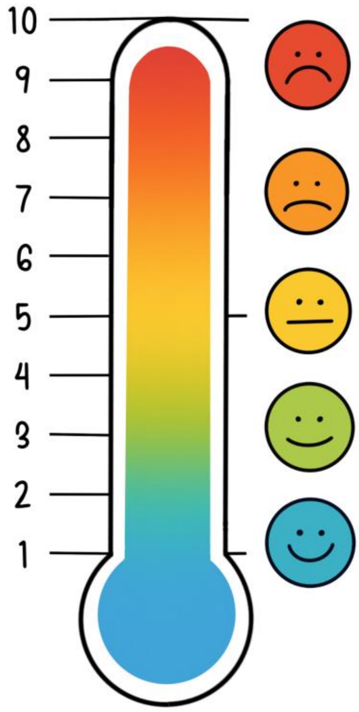

Funded by the European Union

**Name:**	**Date:**

Here we go!

How anxious have you been?

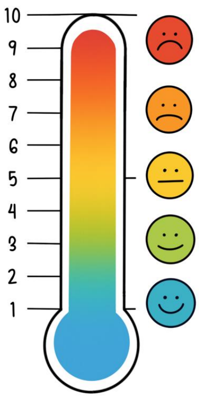

Very anxious

Not anxious at all

How sad have you been?

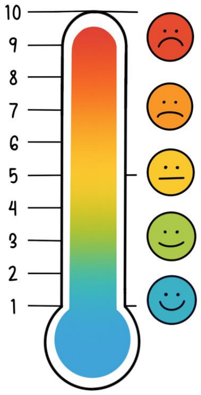

Very sad

Not sad at all

How worried have you been?

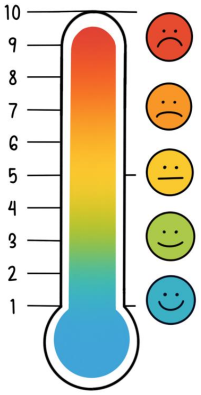

Very worried

Not worried at all

Funded by
the European Union

**Name:**	**Date:**

Great!

Please distribute the balls now.
This will help you to tell me how often something has happened in the last 2 weeks.
Zero balls mean: this has not happened.
Five balls mean: this has happened very often.

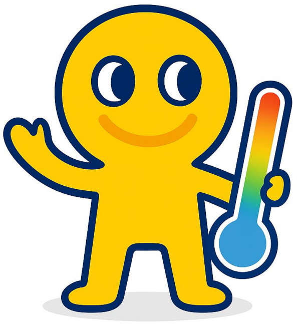

	**Never**	**Almost never**	**Sometimes**	**Often**	**Almost always**
**I feel tired**	☐ Never	☐ Almost never	☐ Sometimes	☐ Often	☐ Almost always
**I feel weak**	☐ Never	☐ Almost never	☐ Sometimes	☐ Often	☐ Almost always
**I sleep a lot**	☐ Never	☐ Almost never	☐ Sometimes	☐ Often	☐ Almost always
**It´s hard for me to concentrate**	☐ Never	☐ Almost never	☐ Sometimes	☐ Often	☐ Almost always

Funded by
the European Union

**Name:**	**Date:**

You have done a great job!

Please distribute the balls now.
This will help you to tell me how often something has happened in the last 2 weeks.
Zero balls mean: this has not happened.
Five balls mean: this has happened very often.

Again, there are thermometers alongside the questions.
Please tick here how bad the situation was for you.

- **A 10 means: it was really bad.**
- **A 1 means: it wasn't bad at all.**

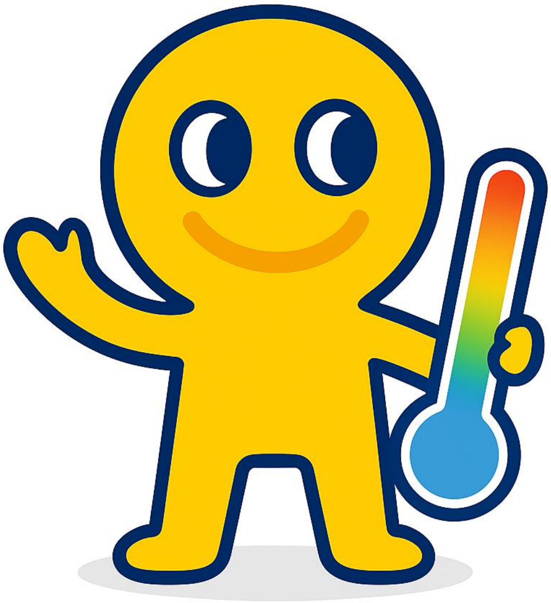

Funded by
the European Union

**Name:**	**Date:**

I am in pain.

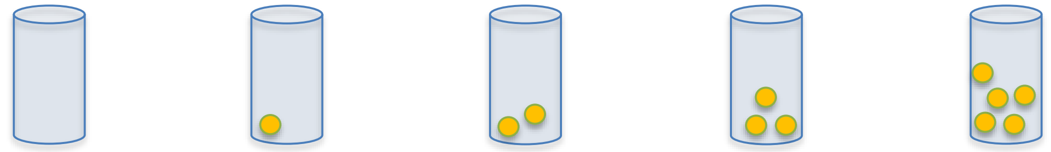

☐ **Never** ☐ **Almost never** ☐ **Sometimes** ☐ **Often** ☐ **Almost always**

For me this is:

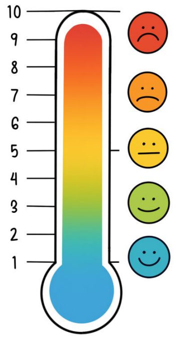

Very bad

Not bad at all

There are arguments in my family.

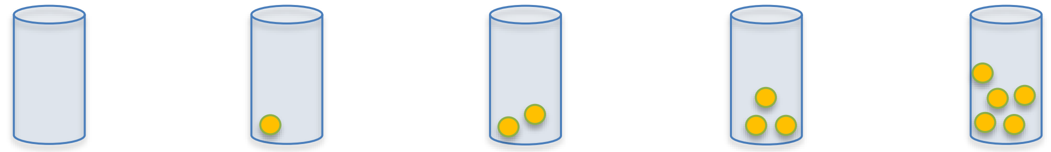

☐ **Never** ☐ **Almost never** ☐ **Sometimes** ☐ **Often** ☐ **Almost always**

For me this is:

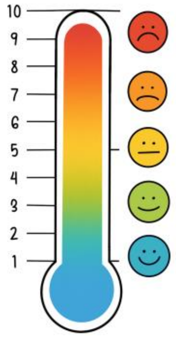

Very bad

Not bad at all

Funded by the European Union

Name:	Date:

I am worried about school (or nursery/kindergarten).

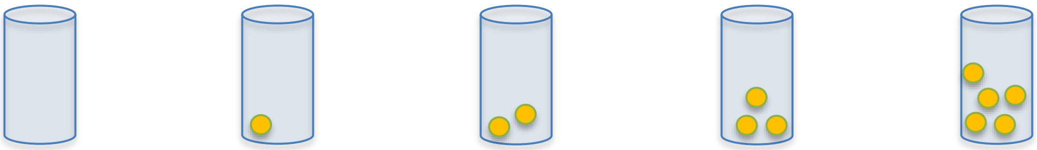

☐ **Never** ☐ **Almost never** ☐ **Sometimes** ☐ **Often** ☐ **Almost always**

For me this is:

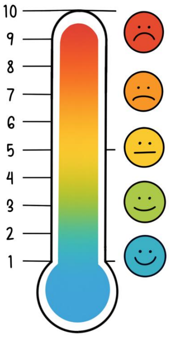

Very bad

Not bad at all

I am worried about my illness.

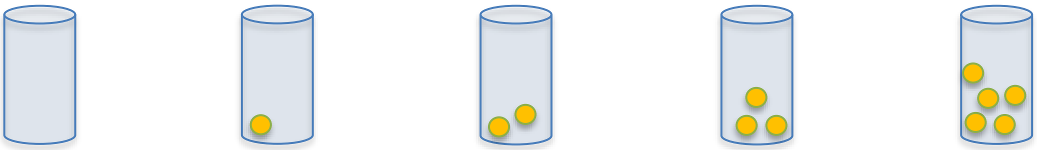

☐ **Never** ☐ **Almost never** ☐ **Sometimes** ☐ **Often** ☐ **Almost always**

For me this is:

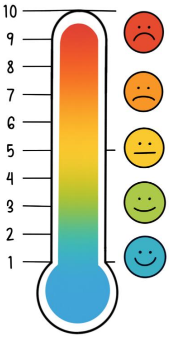

Very bad

Not bad at all

Funded by
the European Union

Name:	Date:

I have trouble with other children.

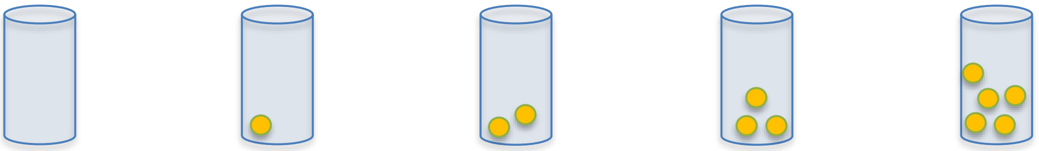

☐ Never ☐ Almost never ☐ Sometimes ☐ Often ☐ Almost always

For me this is:

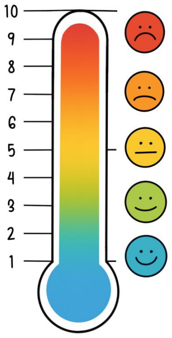

Very bad

Not bad at all

I find it difficult to talk about my illness.

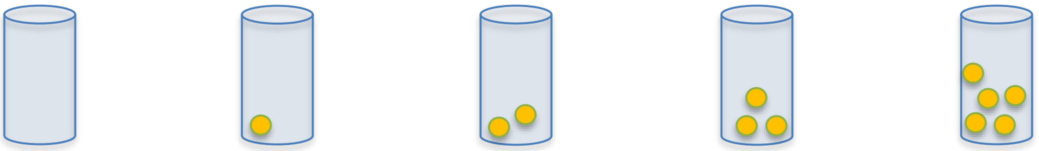

☐ Never ☐ Almost never ☐ Sometimes ☐ Often ☐ Almost always

For me this is:

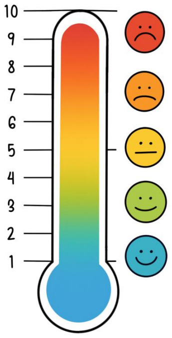

Very bad

Not bad at all

Funded by
the European Union

Name:	Date:

UNIVERSITÄTSmedizin. MAINZ

Zentrum für Kinder- und Jugendmedizin

Mainz Resilience Assessment in Childhood Cancer

MRAcc

For children aged 12 – 17 years

Version 1.0

© 2025, University Medical Center Mainz of the Johannes Gutenberg-University Mainz, Neu, MA., Ortmüller, F., Robinson, AL., Dreismickenbecker, E., Otto, H., Wypyrsczyk, L., Kühn M., Wessa, M., Tüscher, O., Faber, J. All rights are reserved.

Funded by
the European Union

Name:	Date:

1) "Emotions & Stress" Thermometer

Please mark a number from 1 to 10 with an X on each of the following thermometers.

This number shows how strongly you have felt a feeling or a burden in the past 2 weeks, including today.

How anxious have you been?

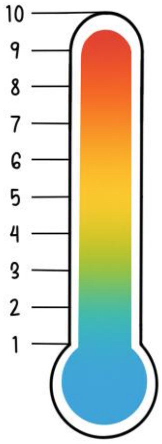

Extremly anxious

Not anxious at all

How sad or depressed have you been?

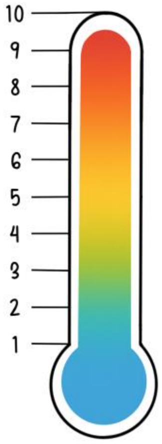

Extremly sad or depressed

Not sad or depressed at all

How worried have you been?

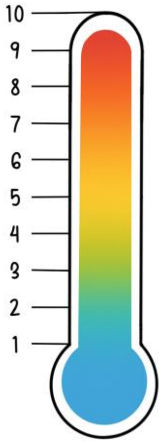

Extremly worried

Not worried at all

Funded by
the European Union

**Name:**	**Date:**

2) "Fatigue- Questions"

Please mark with an **X** how often the following situations occurred in the past 2 weeks (including today)

	Never	Almost never	Sometimes	Often	Almost always
**I feel tired**	☐ Never	☐ Almost never	☐ Sometimes	☐ Often	☐ Almost always
**I feel weak**	☐ Never	☐ Almost never	☐ Sometimes	☐ Often	☐ Almost always
**I sleep a lot**	☐ Never	☐ Almost never	☐ Sometimes	☐ Often	☐ Almost always
**It´s hard for me to concentrate**	☐ Never	☐ Almost never	☐ Sometimes	☐ Often	☐ Almost always

Funded by
the European Union

Name:	Date:

3) "Situations & Experienced Stress" Thermometer

Please mark with an **X** how often the following situations occurred in the past 2 weeks (including today) and to what extent these situations have bothered you.

I am in pain.

- ☐ Never
- ☐ Almost never
- ☐ Sometimes
- ☐ Often
- ☐ Almost always

For me, this is...

Extremly burdensome

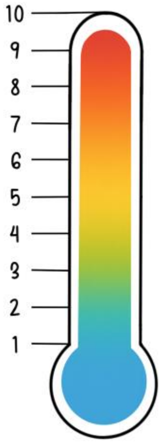

Not burdensome at all

There are arguments in my family.

- ☐ Never
- ☐ Almost never
- ☐ Sometimes
- ☐ Often
- ☐ Almost always

For me, this is...

Extremly burdensome

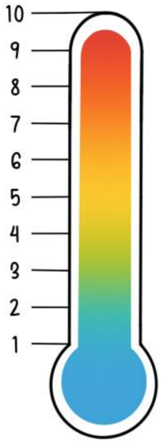

Not burdensome at all

I am worried about school or my education.

- ☐ Never
- ☐ Almost never
- ☐ Sometimes
- ☐ Often
- ☐ Almost always

For me, this is...

Extremly burdensome

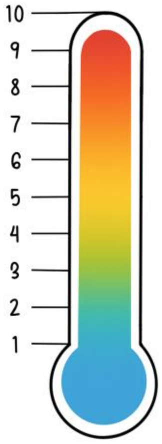

Not burdensome at all

Funded by
the European Union

**Name:**	**Date:**

I am worried about my illness.

☐ **Never**

☐ **Almost never**

☐ **Sometimes**

☐ **Often**

☐ **Almost always**

For me, this is...

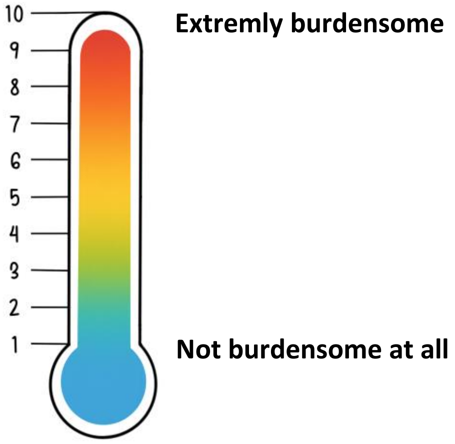

I have trouble with others.

☐ **Never**

☐ **Almost never**

☐ **Sometimes**

☐ **Often**

☐ **Almost always**

For me, this is...

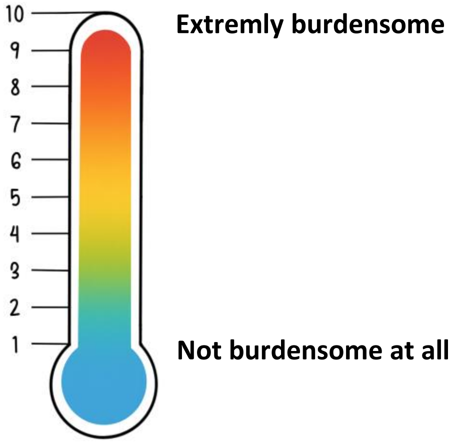

I find it difficult to talk about my illness.

☐ **Never**

☐ **Almost never**

☐ **Sometimes**

☐ **Often**

☐ **Almost always**

For me, this is...

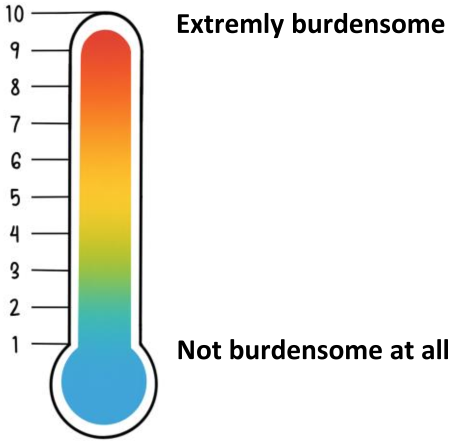

Funded by the European Union

Name:	Date:

UNIVERSITÄTSmedizin. MAINZ

Zentrum für Kinder- und Jugendmedizin

Mainz Resilience Assessment in Childhood Cancer

MRAcc

For children aged 18 + years

Version 1.0

© 2025, University Medical Center Mainz of the Johannes Gutenberg-University Mainz, Neu, MA., Ortmüller, F., Robinson, AL., Dreismickenbecker, E., Otto, H., Wypyrsczyk, L., Kühn M., Wessa, M., Tüscher, O., Faber, J. All rights are reserved.

Funded by
the European Union

Name:	Date:

1) "Emotions & Distress" Thermometer

Please mark a number from 1 to 10 with an X on each of the following thermometers.

This number shows how strongly you have felt a feeling or a burden in the past 2 weeks, including today.

How anxious have you been?

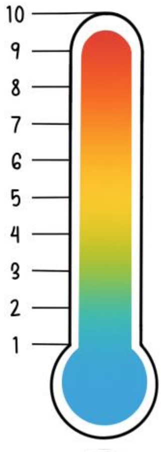

Extremly anxious

Not anxious at all

How sad or depressed have you been?

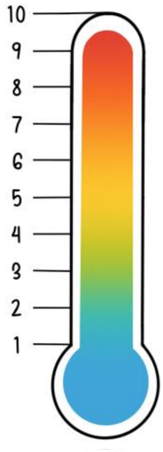

Extremly sad or depressed

Not sad or depressed at all

How worried have you been?

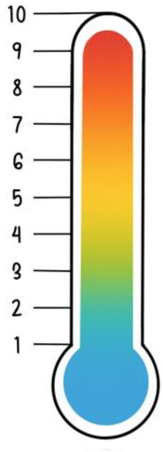

Extremly worried

Not worried at all

Funded by
the European Union

**Name:**	**Date:**

2) Fatigue-Questions"

Please mark with an **X** how often the following situations occurred in the past 2 weeks (including today)

	**Never**	**Almost never**	**Sometimes**	**Often**	**Almost always**
**I feel tired**	☐ Never	☐ Almost never	☐ Sometimes	☐ Often	☐ Almost always
**I feel weak**	☐ Never	☐ Almost never	☐ Sometimes	☐ Often	☐ Almost always
**I sleep a lot**	☐ Never	☐ Almost never	☐ Sometimes	☐ Often	☐ Almost always
**It´s hard for me to concentrate**	☐ Never	☐ Almost never	☐ Sometimes	☐ Often	☐ Almost always

Funded by
the European Union

Name:	Date:

3) "Situations & Experienced Stress" Thermometer

Please mark with an **X** how often the following situations occurred in the past 2 weeks (including today) and to what extent these situations have bothered you.

I am in pain.

- ☐ Never
- ☐ Almost never
- ☐ Sometimes
- ☐ Often
- ☐ Almost always

For me, this is...

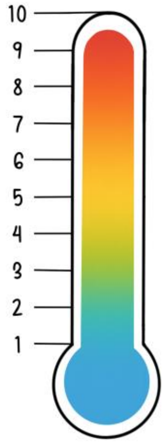

Very burdensome

Not burdensome at all

There are arguments in my family.

- ☐ Never
- ☐ Almost never
- ☐ Sometimes
- ☐ Often
- ☐ Almost always

For me, this is...

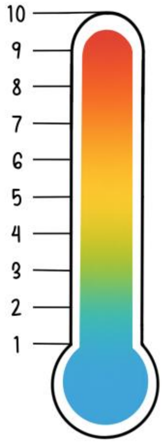

Very burdensome

Not burdensome at all

I am worried about school or professional training or about my work.

- ☐ Never
- ☐ Almost never
- ☐ Sometimes
- ☐ Often
- ☐ Almost always

For me, this is...

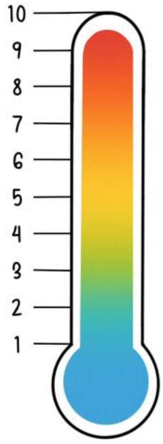

Very burdensome

Not burdensome at all

Funded by
the European Union

Name:	Date:

I am worried about my illness.

- ☐ Never
- ☐ Almost never
- ☐ Sometimes
- ☐ Often
- ☐ Almost always

For me, this is…

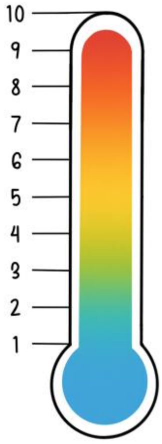

Extremly burdensome

Not burdensome at all

I have trouble with others.

- ☐ Never
- ☐ Almost never
- ☐ Sometimes
- ☐ Often
- ☐ Almost always

For me, this is…

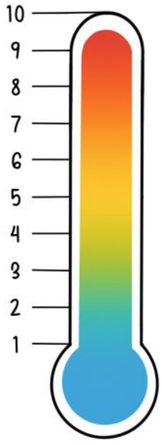

Extremly burdensome

Not burdensome at all

I find it difficult to talk about my illness.

- ☐ Never
- ☐ Almost never
- ☐ Sometimes
- ☐ Often
- ☐ Almost always

For me, this is…

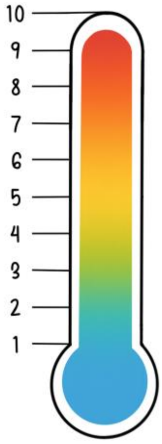

Extremly burdensome

Not burdensome at all

Funded by
the European Union
