# Supplementary material for: The mainz resilience assessment in childhood cancer (MRAcc): development of a novel age-specific patient-reported outcome measure to assess resilience in childhood cancer patients
Source: BMC Cancer. 2026 Feb 26;26:312. doi: 10.1186/s12885-026-15776-y (PMC12952046; doi:10.1186/s12885-026-15776-y)
Supplement: Supplementary file 2 — Additional file 2: Missing data rules [file 12885_2026_15776_MOESM2_ESM.pdf]

Supplementary File S2:

Missing Data Rules for MRAcc Score Computation

Overview

This document provides the predefined rules for handling incomplete questionnaire responses in the Mainz Resilience Assessment in Childhood Cancer (MRAcc). These rules were established prior to data analysis to ensure transparent, consistent and reproducible score calculation.

1. Mental Health Score

For the mental health score, missingness will be addressed separately for the emotion and distress thermometers and for the fatigue subscale.

For the **emotion and distress thermometers**, if one thermometer item is missing, the subscore should be calculated by averaging across the remaining items. If two or more thermometer items are missing, no mental health score should be computed.

For the **fatigue subscale**, the score should be calculated if at least three of the four items are answered. If more than one item is missing, the fatigue subscale should not be derived, and the mental health score can only be calculated if all three thermometer items are available.

In summary, a **mental health score** should only be computed if at least three of the four dimensions (anxiety, depression, distress, fatigue) are available.

2. Stressor Exposure Score

For the stressor exposure score, missingness will be handled at the level of **frequency–intensity pairs**.

If the intensity item is missing but the corresponding frequency is available, the intensity value

should be set to 1 as the lowest possible rating. Conversely, if the frequency item is missing but the corresponding intensity is available, the frequency value should be set to 1.

If both items of a stressor dimension are missing, this dimension cannot be calculated. The **stressor exposure score** should be derived if at least four of the six stressor dimensions are available, with the final score based on the number of completed dimensions. If more than two stressor dimensions are missing, no stressor exposure score should be calculated.

3. MRAcc Stressor Reactivity Score

The overall **MRAcc stressor reactivity score** should be only derived if both component scores, the mental health score and the stressor exposure score, are available.

If either component cannot be calculated, the overall MRAcc stressor reactivity score should be set to missing.
